# DNA damage and repair in patients with early chronic kidney disease with or without type 2 diabetes

**DOI:** 10.3389/fcdhc.2025.1601311

**Published:** 2025-09-17

**Authors:** Jorge Andrade-Sierra, Leonardo Pazarín-Villaseñor, Andrés García-Sánchez, Ernesto Germán Cardona-Muñoz, Wendy Campos-Pérez, Erika Martínez-López, Tannia Isabel Campos-Bayardo, Daniel Román-Rojas, Luis Francisco Gómez-Hermosillo, Jorge Casillas-Moreno, Raquel Echavarría, Elodia Nataly Díaz-de la Cruz, Sylvia Totsuka-Sutto, Alejandra Guillermina Miranda-Díaz

**Affiliations:** ^1^ Nephrology Service, Civil Hospital of Guadalajara “Dr. Juan I Menchaca”, Guadalajara, Jalisco, Mexico; ^2^ Department of Physiology, University Center of Health Sciences, University of Guadalajara, Guadalajara, Jalisco, Mexico; ^3^ Department of Molecular Biology and Genomics, Institute of Nutrigenetics and Translational Nutrigenomics, University of Guadalajara, Guadalajara, Jalisco, Mexico; ^4^ Department of Surgery, Civil Hospital of Guadalajara “Dr. Juan I Menchaca”, Guadalajara, Jalisco, Mexico; ^5^ Secihti- University Center of Los Altos, University of Guadalajara, Tepatitlán de Morelos, Jalisco, Mexico

**Keywords:** oxidative damage to DNA, repair of oxidative damage to DNA, early kidney disease, chronic kidney disease, diabetes mellitus

## Abstract

**Introduction:**

Chronic kidney disease (CKD) may improve with appropriate management and close monitoring to prevent the risk of progression to end-stage kidney disease (ESKD). The present study aimed to determine oxidative damage and DNA repair in early kidney disease in patients with and without type 2 diabetes (T2D).

**Methods:**

Using ELISA, serum levels of the oxidative DNA damage marker (8OHdG) and the DNA repair marker (hOGG1) were determined in 100 patients with T2D and 88 without T2D in stages 1, 2, and 3 of CKD.

**Results:**

The mean number of years of T2D in patients in stages 1, 2, and 3 was 13.93 ± 2.09 years. Significantly increased levels of the 8-OHdG marker were found in stage 3 CKD patients with T2D, 4.96(4.17-5.08) ng/mL *vs*. 4.13(3.49-4.60) ng/mL without T2D (*p*=0.006). hOGG1 enzyme levels were significantly decreased in patients with T2D from stage 2, 0.08(0.063-0.082) ng/mL *vs*. 0.37(0.18-0.36) ng/mL, (*p*=0.006) and in stage 3 with T2D 0.09(0.08-0.11) ng/mL *vs*. 0.53(0.07-0.96) ng/mL without T2D (*p*=0.007). A positive correlation was found between CKD stage and hOGG1 levels in patients with T2D (rho=0.473, *p*<0.001). 8-OHdG concentration showed an inverse correlation with CKD stage in patients without T2D (rho=-0-274, *p*=0.030). In conclusion, we found an imbalance of DNA repair enzymes in stages 2 and 3 of CKD in T2D patients and an increase of oxidative DNA damage markers in stage 3 of CKD in T2D patients. Determination of DNA damage and repair markers in the early stages of CKD may facilitate timely diagnosis and treatment of CKD.

## 1 Introduction

Chronic kidney disease (CKD) is a common condition characterized by evidence of kidney damage or dysfunction (1). Type 2 diabetes (T2D) and high blood pressure are the main causes of up to two-thirds of CKD cases (2). Other less common causes capable of producing CKD include glomerulonephritis, nephrolithiasis, infections, environmental exposures (air pollution, pesticides), and polycystic kidney disease (3). CKD is defined as a GFR <60 mL/min/1.73 m² and/or an albumin-to-creatinine ratio (ACR) ≥30 mg/g [3 mg/mmol], or other markers of kidney damage (4). The early stages of CKD may be asymptomatic due to the lack of obvious clinical symptoms. Symptoms become evident when severe damage has already occurred. CKD is divided into 5 stages according to the eGFR (5).

Early detection, diagnosis, and management of the disease allow active intervention to slow the progression of kidney disease and metabolic complications to reduce cardiovascular morbidity (6). The CKD progression rate varies in patients depending on the etiology and pathology that caused the disease (7). T2D is a heterogeneous pathology characterized by hyperglycemia resulting from defects in insulin action/secretion or both (8). T2D can produce microvascular complications in the kidney, leading to diabetic nephropathy (DN). DN is the most common complication of T2D and is the main cause of end-stage kidney disease (ESKD), which produces high morbidity and mortality (9). The first changes due to DN are hemodynamic, starting with glomerular hyperperfusion, favoring the initial increase in GFR manifested by hyperfiltration. This hyperfiltration occurs parallel to kidney hypertrophy, leading to increased serum creatinine and albumin leakage from the glomerular capillaries. However, increased serum creatinine may not be present in some patients in the early stages of CKD (10, 11).

The oxidative stress (OS) plays an important role in cardiovascular morbidity and mortality in advanced CKD (12). The marker 8-hydroxy-2-deoxyguanosine (8-OHdG) is one of the most abundant oxidative products of DNA, it is a sensitive biomarker of OS capable of reflecting oxidative DNA damage. Serum 8-OHdG serves as a surrogate marker of oxidant-induced DNA damage in dialysis patients. However, the predictive capacity of 8-OHdG for mortality in patients with CKD in earlier stages is poorly studied (13). The main enzyme that repairs oxidative DNA damage is 8-oxoguanine glycosylase (hOGG1), whose predominant function occurs through base excision mechanisms (14). Reduced hOGG1 activity heightens cellular vulnerability to mutagenic insults from toxic exposures such as tobacco smoke, alcohol, and uremic toxins (14).

The purpose of the study was to determine oxidative damage and DNA repair in early kidney disease in patients with and without T2D.

## 2 Materials and methods

A cross-sectional analytical study was conducted in patients diagnosed with early-stage CKD (1, 2, 3) with and without T2D treated in the Nephrology Department of the Hospital Civil de Guadalajara “Juan I. Manchaca” in Guadalajara, Jalisco, Mexico, who agreed to participate in the study and signed the informed consent. Patients of both sexes over 18 years of age were included. Patients who had taken antioxidants (vitamin E, vitamin C, etc.) three months before the study, patients with clinical or biochemical signs of an infectious process, or those on renal replacement therapy (RRT) were not included. Anthropometric, clinical and biochemical data were measured: body mass index (BMI Kg/m^2^), systolic blood pressure (SBP) and diastolic blood pressure (DBP), hemoglobin (Hb), hematocrit, glucose, glycated hemoglobin (HbA1c), creatinine, total cholesterol, high-density cholesterol, low-density cholesterol, triglycerides, albumin, sodium, potassium, chloride, calcium, phosphorus, urine glucose, 24-h urine protein and GFR.

5 mL of venous blood was obtained in a test tube containing ethylenediaminetetraacetic acid (EDTA) to obtain plasma and 5 mL in a dry tube to obtain serum. Plasma and serum were centrifuged at 1800 revolutions for 10 min (rpm), then the samples were stored at -80°C until final processing.

### 2.1 Causes of early kidney disease in non-T2D patients

Causes of CKD include early kidney inflammation, tubulointerstitial injury, glomerulosclerosis, polycystic disease, and other unidentified (15). The causes of early kidney disease in non-T2D patients were predominantly glomerulonephritis, polycystic disease, and nephrotoxic substances.

### 2.2 Oxidative DNA damage

Serum levels of 8-hydroxy-2-deoxyguanosine were measured using the ELISA kit MBS267161 (MyBioSource^®^). The kit was kept at 4°C initially and then at room temperature. For the standard curve, 1.0 mL of standard diluent was added to the vial of lyophilized standard and allowed to stand for 30 min. The standard was completely dissolved according to the manufacturer’s instructions. 100 µL of the standard or samples was added to the wells. The 96-well plate was sealed and incubated at 37°C for 90 min. It was washed twice, 100 µL of biotinylated antibody was added, and the plate was incubated at 37°C for 60 min. It was washed three times, and 100 µL of enzyme conjugate was added. The plate was incubated for 30 min at 37°C and washed five times. 100 µL of color reagent was added, and the plate was incubated in the dark at 37°C. The chromogenic reaction was monitored for 30 min or less, depending on the coloration of the standards. The reaction was stopped by adding 100 µL of color reagent C to each well. The plate was read at an optical density of 450 nm. The measurements were performed in duplicate.

### 2.3 Oxidative DNA repair

It was performance by the manufacturer MyBioSource^®^ h8-oxoguanine DNA glycosidase (hOGG1) Elisa kit no. MBS702793. It was a sandwich ELISA. We added 100 μL of sample or standard in a 96-well plate pre-coated with and specific antibody for hOGG1 and incubated for 1 h at 37°C. After washing the plate, 2 more incubation were followed with biotin-conjugated antibody specific for hOGG1 and HRP. A color signal was obtained by adding TMB substrate. The color development was measured at 450 nm. Two measurements of each biomarker were performed. The measurements were performed in duplicate.

### 2.4 Statistical analysis

Qualitative results are expressed as frequency ratios. Quantitative data are shown as mean and standard error of the mean (SEM). Confidence intervals are included for markers of oxidative damage. The Kolmogorov-Smirnov test was used to determine the normality of data for stages 2 and 3, and the Shapiro-Wilk test was used for stage 1, depending on the sample size. Comparative analysis of parametric and nonparametric data was performed using the independent samples Student *t*-test and the Mann-Whitney U test, respectively. The False Discovery Rate (Benjamini-Hochberg) test was used as an adjustment for significant results from multiple analyses. Spearman correlation coefficient was estimated to determine the linear association between variables. All results with a p-value ≤0.05 were considered statistically significant.

### 2.5 Ethical considerations

The study adhered to the ethical principles for medical research involving human subjects stipulated in the Declaration of Helsinki 64th General Assembly Fortaleza, Brazil, October 2013, in the Belmont Report and Standards of Good Clinical Practice according to the guidelines of the International Conference on Harmonization by the provisions of the General Health Law by the Regulation on Health Research art. 17. The study corresponds to category II (minimal risk research). A consent letter was required under Information. The study was authorized by the Research and Ethics Committee of the Civil Hospital of Guadalajara “Juan I. Manchaca” with State Registry 069/15 HCJM/2015.

## 3 Results

This study included 100 patients with T2D and 88 patients without T2D. Of these, 81 were men and 107 were women. The mean age of the patients was 51.77 ± 22.02 years. The duration of T2D in stage 1 patients was 9.8 ± 2.52 years, in stage 2, 16.00 ± 1.59 years and in stage 3, 16.13 ± 1.33 years. 13 patients without T2D and seven with T2D consumed alcohol. 38 patients without T2D were smokers and 55 with T2D. Eight patients without T2D were in stage 1, 34 in stage 2 and 46 in stage 3. Of the patients with T2D, 10 were in stage 1, 37 in stage 2 and 53 in stage 3. Body mass index (BMI) and SBP and DBP values ​​were similar in all included patients. Hyperglycemia was significant in all three stages of kidney disease in patients with T2D, (stage 1, 149.50 ± 19.63 mg/dL vs. without T2D, 90.50 ± 3.13 mg/mL (p=0.02), stage 2, 158.87 ± 10.29 mg/dL vs. without T2D 94.02 ± 2.76 mg/dL (p=0.0001) and in stage 3, 144.18 ± 9.68 mg/dL vs. 93.40 ± 3.44 mg/dL without T2D) (p=0.0001). HbA1c was found to be increased in patients with T2D and early kidney disease (stage 1, 8.77 ± 0.53%, stage 2, 8.31 ± 0.38% and stage 3, 8.43 ± 0.14%) compared to patients without T2D (stage 1, 5.20 ± 0.83%, stage 2, 5.54 ± 0.09% and stage 3, 5.33 ± 0.14%) (p=0.0001). Total serum proteins had higher levels in patients with stage 2 T2D with 153.50 ± 21.59 g/dL vs. without T2D 92.08 ± 18.85 g/dL (p=0.04). Serum albumin levels were higher in patients with stage 2 T2D, 3.89 ± 0.07 g/dL vs. without T2D, 3.57 ± 0.08 g/dL (p=0.003). Triglycerides were slightly elevated in patients with stage 3 T2D, 193.08 ± 14.86 mg/dL vs. 152.92 ± 9.61 mg/dL in patients without T2D (p=0.03). Serum creatinine was found to be higher in stage 3 patients without T2D, 1.65 ± 0.06 mg/dL vs. 1.44 ± 0.04 mg/dL (p=0.005). Glycosuria was present in patients with T2D, stage 1, 462.50 ± 124.75 mg/dL, stage 2, 300.89 ± 38.96 mg/dL and stage 3, 323.71 ± 37.72 mg/dL. GFR was found to decrease progressively according to the stage of the disease. GFR in stage 1 T2D patients was lower 100.4 ± 3.44 mL/min/1.73 m2 vs. 115.13 ± 5.09 mL/min/1.73 m2 (p=0.02) and patients without T2D in stage 3 showed lower GFR, 41.78 ± 1.26 mL/min/1.73 m2 compared to patients with T2D, 45.45 ± 1.23 mL/min/1.73 m2 (p=0.04). **Table 1**.

**Table 1:** Early chronic kidney disease in patients with and without T2D.

	Stage 1		*p*	Stage 2		*p*	Stage 3		*p*
	T2D N-10	No T2D N-8		T2D N-37	No T2D N-34		T2D N-53	No T2D N-46	
Anthropometric and clinical data									
Gender F/M	8/2	4/4		17/20	16/18		33/20	29/17	
Age years	50.7 ± 5.81	28.00 ± 2.90	0.005*^T^	65.14 ± 1.78	55.38 ± 3.57	0.04**	61.40 ± 1.96	50.00 ± 3.30	0.024**
BMI kg/m^2^	29.91 ± 1.62	25.60 ± 1.45	0.07	28.94 ± 0.99	28.97 ± 1.00	0.86	28.94 ± 0.99	28.27 ± 0.99	0.58
DBP mm/Hg	73.90 ± 3.18	78.00 ± 4.69	0.47	77.46 ± 1.74	76.15 ± 2.17	0.64	77.60 ± 1.97	81.91 ± 2.33	0.16
SBP mm/Hg	128.50 ± 5.60	129.29 ± 2.97	0.91	127.86 ± 3.19	126.09 ± 3.75	0.72	132.02 ± 3.43	128.96 ± 3.36	0.52
Biochemical data									
T2D years	9.8 ± 2.52			16.00 ± 1.59			16.13 ± 1.33		
Blood glucose mg/dL	149.50 ± 19.63	90.50 ± 3.13	0.02*	158.87 ± 10.29	94.02 ± 2.76	0.0001**^T^	144.18 ± 9.68	93.40 ± 3.44	0.0001**^T^
HbA1c %	8.77 ± 0.53	5.20 ± 0.83	0.001*^T^	8.31 ± 0.38	5.54 ± 0.09	0.0001*	8.43 ± 0.34	5.33 ± 0.14	0.001**
Calcium mg/dL	9.18 ± 0.42	8.86 ± 0.43	0.13	9.07 ± 0.08	8.94 ± 0.22	64	8.92 ± 0.07	8.97 ± 0.08	0.64
Total proteins g/dL	93.75 ± 32.55	192.00 ± 52.29	0.11	153.50 ± 21.59	92.08 ± 18.85	0.04*	98.45 ± 14.53	115.00 ± 14.79	0.76
Albumin g/dL	2.65 ± 0.42	2.95 ± 0.83	0.73	3.89 ± 0.07	3.57 ± 0.08	0.02**	3.76 ± 0.15	3.57 ± 0.09	0.30
Total cholesterol mg/dL	169.67 ± 26.36	173.40 ± 10.92	0.90	177.50 ± 6.56	185.89 ± 9.46	0.46	176.55 ± 6.47	179.32 ± 5.33	0.75
Triglycerides mg/dL	188.11 ± 39.48	158.00 ± 35.51	0.59	178.56 ± 13.04	161.04 ± 13.01	0.35	193.08 ± 14.86	152.92 ± 9.61	0.03*
HLD mg/dL	47.40 ± 4.80	38.78 ± 0.77	0.13	38.60 ± 1.41	40.95 ± 2.00	0.33	42.09 ± 1.54	44.58 ± 2.09	0.33
LDL mg/dL	86.31 ± 16.80	97.58 ± 10.16	0.11	103.33 ± 6.18	109.47 ± 8.45	0.55	91.98 ± 5.08	103.09 ± 4.04	0.09
Renal function data									
Creatinine mg/dL	0.70 ± 0.05	0.77 ± 0.07	0.42	0.96 ± 0.03	1.02 ± 0.03	0.18	1.44 ± 0.04	1.65 ± 0.06	0.005**^T^
Phosphorus mg/dL	3.48 ± 0.16	3.90 ± 0.18	0.10	3.42 ± 0.11	3.61 ± 0.15	0.31	3.68 ± 0.09	3.55 ± 0.10	0.33
Potassium mEq/L	4.23 ± 0.20	4.01 ± 0.10	0.37	4.53 ± 0.11	4.38 ± 0.11	0.34	4.71 ± 0.11	4.60 ± 0.08	0.43
Urinary protein mg/24h	154.68 ± 64.95	116.06 ± 43.81	0.65	82.50 ± 22.97	72.81 ± 13.55	0.72	103.67 ± 23.91	97.61 ± 15.89	0.84
Urinary glucose mg/dL	462.50 ± 124.75	(-)		300.89 ± 38.96	(-)		323.71 ± 37.72	(-)	
GFR mL/min/1.73 m^2^	100.4 ± 3.44	115.13 ± 5.09	0.02*	74.92 ± 1.39	74.26 ± 1.49	0.74	45.45 ± 1.23	41.78 ± 1.26	0.04**

F, female; M, Male; T2D, type 2 diabetes; BMI, body mass index; SBP, systolic blood pressure; DBP, diastolic blood pressure; HbA1c, glycated hemoglobin; HDL, high density cholesterol; LDL, low density cholesterol; GFR, glomerular filtration rate. Values are expressed as mean ± standard error (SE). *statistically significant using *t*-Student test of independent samples; **statistically significant using the Mann-Whitney U-test; ^T^statistically significant using False Discovery Rate (Benjamini-Hochberg) test.

**Table 2** shows that the levels of the marker of oxidative DNA damage in stages 1 and 2 of patients with and without T2D were similar. In stage 3 of early kidney disease, an increase in the marker 8-OHdG was found in patients with T2D 4.96(4.17-5.08) ng/mL *vs*. 4.13(3.49-4.60) ng/mL (*p*=0.006) in patients without T2D. The DNA repair marker hOGG1 was found to be significantly decreased in patients with stage 2 T2D, 0.08(0.063-0.082) ng/mL *vs*. 0.37(0.18-0.36) ng/mL in patients without T2D (*p*=0.006). The same phenomenon was found in stage 3 T2D where the levels of the oxidative damage repair marker to DNA showed 0.09(0.08-0.11) ng/mL *vs*. 0.53(0.07-0.96) ng/mL of patients without T2D (*p*=0.007).

**Table 2:** DNA damage and repair marker in early kidney disease with and without T2D.

	Stage 1		*p*	Stage 2		*p*	Stage 3		*p*
	T2D	No T2D		T2D	No T2D		T2D	No T2D	
h8-OHdG ng/mL	5.14 (3.62-6.67)	5.19 (3.59-7.03)	0.95	4.77 (4.47-5.33)	4.67 (4.08-4.97)	0.75	4.96 (4.17-5.08)	4.13 (3.49-4.60)	0.006*
hOGG1] ng/mL	0.05 (0.03-0.065)	0.68 (-0.25-1.44)	0.07	0.08 (0.063-0.082)	0.37 (0.18-0.36)	0.006*	0.09 (0.08-0.11)	0.53 (0.07-0.96)	0.007*

DNA, deoxyribonucleic acid; T2D, type 2 diabetes; h8-OHdG, human 8 hydroxy-2-deoxy guanosine; hOGG1, human oxyguanosine. Values are expressed as mean and (Confidence intervals). *statistically significant using the Mann-Whitney U-test, *p ≤* 0.05

**Figure 1:**
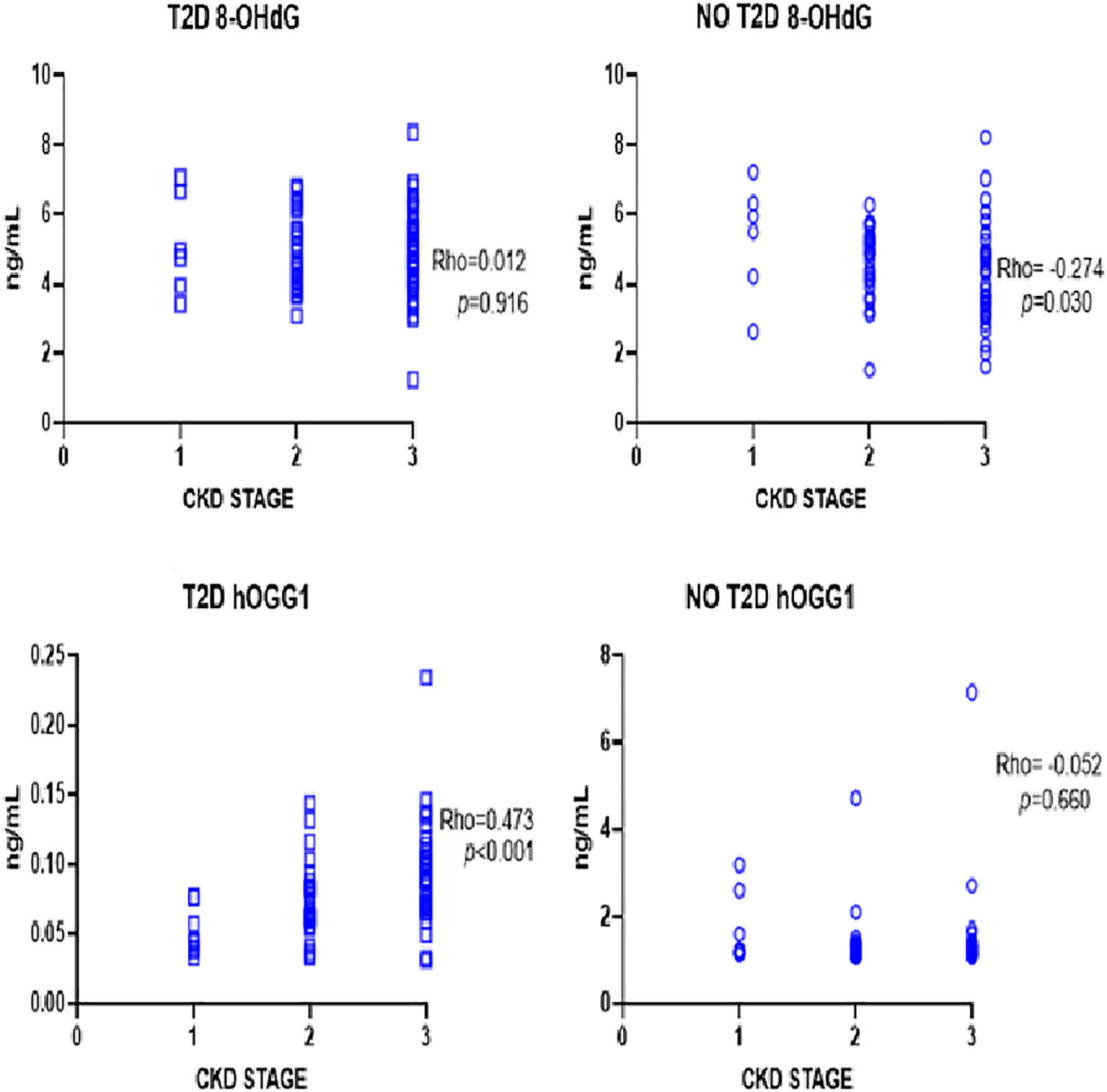
Correlation analysis between dna damage and repair markers in early renal disease. Increased hOGG1 correlates positively with increasing CKD stage in T2D patients. Decreased 8-OHdG correlates with increasing CKD stage.

A correlation analysis was performed to identify associations between levels of oxidative DNA damage, the repair enzyme, and CKD progression. An increase in hOGG1 concentration was directly proportional to CKD stage progression in patients with T2D (rho=0.473, *p*<0.001). On the other hand, patients without T2D showed no significant correlation between hOGG1 levels and CKD stage (rho=-0.052, *p*=0.660). Oxidative DNA damage showed an inverse correlation between 8-OHdG levels and CKD progression in patients without T2D (rho=-0.274, *p*=0.030). However, patients with T2D showed no correlation between oxidative DNA damage and CKD stage progression (rho=0.012, *p*=0.916). **Figure 1**.

Spearman correlation analysis between CKD phase and oxidative stress markers. CKD = chronic kidney disease, ND = Non- T2D = type 2 diabetes, hOGG1 = 8-oxoguanine glycosylase, 8-OHdG=8-hydroxy-2-deoxyguanosine.

## 4 Discussion

Excessive OS plays an important role in the progression of CKD. OS is significantly involved in T2D, hypertension, kidney ischemia, glomerular damage, inflammation, and endothelial dysfunction (16). Elevated serum 8-OHdG levels serve as a surrogate marker of oxidant-induced DNA damage. In the present study, patients with early CKD were included (1, 2, 3). A total of 100 patients with T2D and 88 patients without T2D were evaluated. The levels of the 8-OHdG marker were comparable in stages 1 and 2 of chronic kidney disease (CKD) in both groups. However, in stage 3 CKD, patients with T2D exhibited a significant increase in marker levels compared to those without T2D.

This result is particularly intriguing, as a review of the available scientific literature did not reveal information on the behavior of the 8-OHdG marker in the early stages of CKD. Specifically, no evidence suggests that its increase begins in the initial stages of CKD, nor that a higher index of suspicion is warranted in patients with T2D. After all, higher plasma concentrations of 8-OHdG have been related to a higher risk of developing ESKD (17, 18). The increase and correlation between serum levels of the marker 8-OHdG in stages 4–5 of CKD with eGFR has been published (15) without this event having occurred in the patients included in the present study even though the eGFR was found to be lower in patients with stage 1 T2D and stage 3 without T2D. It should be considered that serum 8-OHdG is associated with a higher risk of all-cause mortality in individuals with a wide range of eGFR and the association is independent of the persistent inflammatory state suffered by the patients as recently published (19).

hOGG1 is an enzyme involved in the DNA repair pathway by base excision mechanisms. In the context of early kidney disease, hOGG1 plays an important role in maintaining genomic stability. The hOGG1 glycosylase enzyme is the first step along the DNA base excision repair pathway. The action of the enzyme ensures the integrity of the genome by preventing the occurrence of mutations (20). It has been reported that alterations in hOGG1 may affect its enzymatic function, which could lead to increased OS and contribute to the progression of kidney disease (21). The mechanism of lesion recognition by the hOGG1 enzyme has received much attention, mainly through the study of the structure of the protein and some mutant derivatives in the presence of DNA harboring 8-oxoG residues. Initial studies led to the proposal of a mechanism by which the enzyme facilitates the extrusion of the damaged base from the interior of the DNA helix due to the disruption of base pairing induced by local DNA bending (22). The trajectory of intra-helical lesion recognition and extrusion by the human 8-oxoguanine DNA glycosylase. hOGG1, which initiates the DNA base excision repair pathway, faces an enormous challenge in finding instances of 8-oxoG among an excess of normal guanines (23). The binding of hOGG1 to an oligonucleotide harboring an intra-helical 8-oxoG provides crucial information about key residues involved in the DNA damage marker extrusion process (24). There are other mechanisms of action of the enzyme. Research has suggested that hOGG1 inhibition could have anti-inflammatory and antifibrotic effects. In addition to repairing damaged DNA, hOGG1 can activate inflammatory signals, such as TNF-α, that contribute to fibrosis and kidney deterioration. Therefore, some studies have explored the possibility of developing hOGG1 inhibitors as a therapeutic strategy for inflammatory diseases and fibrosis, including CKD (25).

In the present study, it is worth noting the significant decrease in the expression of the DNA repair enzyme in patients with stage 2 and 3 CKD and T2D compared to the levels found in patients without T2D. This could condition the progression of CKD, with a predominance of patients with T2D. Therefore, monitoring the activity and expression levels of the enzyme is recommended because its determination can provide information on the state of OS and the degree of DNA damage in patients with early kidney disease with and without T2D.

Other risk factors may influence the development of early CKD, such as age and T2D. Both factors were found in the present study. Patients with all three stages of early kidney disease were older and had T2D. The lifetime risk of developing CKD could not distinguish between early CKD and normal age-related decline in kidney function. The confluence of the two factors mentioned above favors the reduction of blood flow and Kidney mass, and the increase in glomerulosclerosis as part of the normal aging process favors the decrease in GFR of approximately 0.75 mL/min/1.73 m^2^ per year from the age of 40 years (26). It is difficult to differentiate between age-related loss of kidney function and kidney disease *per se* (27). However, there is evidence against defining CKD based on age, although the interaction between kidney function and proteinuria appears to differ with age (28, 29). According to our results, it can be argued that changes in GFR in senescence are due to other pathological processes such as the presence of T2D rather than age-related kidney decline.

In the present study, systolic and diagnostic blood pressure remained within acceptable limits. However, it is important to consider arterial hypertension as a risk factor capable of affecting GFR as has been previously published in multiple scientific reports. High blood pressure is defined as blood pressure (BP) ≥140/80 mmHg. Hypertension affects approximately 30% of the general adult population and up to 90% of people with CKD according to the European Society of Cardiology and the European Society of Hypertension (30). Hypertension is both a cause and an effect of CKD and contributes to its progression (31). As eGFR decreases, the incidence and severity of hypertension increases. Hypertension and chronic kidney disease are independent risk factors for cardiovascular disease (CVD). When both coexist, the risks of CVD morbidity and mortality increase substantially. In people with stage 3 (eGFR 30–59 mL/min/1.73 m^2^) or stage 4 (eGFR 15–29 mL/min/1.73 m^2^) CKD. The risk of death from CVD is greater than the risk of progression to End-Stage Kidney Disease (ESKD) (eGFR <15 mL/min/1.73 m^2^) (32).

Hemoglobin A1c is a crucial indicator of T2D status. In the present study, patients with early CKD stages 1, 2, and 3 had HbA1c levels greater than 8%, which translates to a higher risk of complications and death, as recently reported. Elevated HbA1c levels (8%) increase the risk of death by inducing atrial remodeling, inflammation, and hypercoagulability, which can significantly affect mortality (33).

Patients in the first three stages of CKD included in the present study remained within the overweight range according to other publications (34). Obesity is also an important risk factor for the development and progression of CKD (35). However, current evidence suggests that once CKD is established, obesity is paradoxically associated with increased survival, particularly among those receiving hemodialysis. This phenomenon is referred to as the “obesity paradox” or “reverse epidemiology” (36). Emerging data suggest that weight loss interventions slow or reverse the progression of early CKD (37).

We consider it essential to identify CKD early through specific detection based on known risk factors, followed by risk stratification and individualized treatment, in addition to implementing sensitive biomarkers that can favor early detection of the progression of the disease. The above substantially reduces morbidity and mortality due to CKD and related complications such as cardiovascular disease (38). Prompt and expeditious clinical care can improve in the early stages (1, 2, 3 KDOQI) of the disease through common control measures such as decreased sodium intake, control of T2D, blood pressure, and overweight among others, to prevent progression to ESKD (39). Timely management of early kidney disease in patients with and without T2D is particularly relevant in socially disadvantaged and vulnerable populations.

## 5 Conclusion

Even mildly reduced kidney function predicts a higher risk of premature mortality. The risk increases with the continued progression of CKD, making it imperative to identify and treat CKD in the earliest stages promptly with a high index of suspicion. The evaluation of DNA damage and repair mechanisms is becoming increasingly important because the lack of sensitive and specific biomarkers makes early diagnosis and treatment of kidney disease difficult. In the present study, the levels of the oxidative DNA damage marker were found to be increased in stage 3 of kidney disease in patients with T2D, and a significant decrease in the DNA repair enzyme was even found in patients with T2D in stages 2 and 3.

The assessment of DNA damage and repair markers, such as 8-OHdG and hOGG1, may occur alongside aging processes. However, our findings suggest that changes in GFR during senescence are driven by other pathological factors - most notably, the presence of T2D - rather than by age-related kidney decline or its progression from early stages. Reduced levels of the hOGG1 enzyme could suggest an attempt to compensate for the imbalance of the oxidative DNA damage marker in T2D patients with stage 3 CKD. The study of early CKD and established CKD in patients with and without T2D is an interesting and completely unfinished topic. New biomarkers that may help elucidate the underlying mechanisms of CKD are continually emerging in the scientific literature.

### 5.1 Limitations of the study

The sample collected was obtained through simple randomization from a hospital Nephrology Service in search of patients with and without T2D of different stages of CKD. This resulted in small subgroups with unequal sizes, thus statistically unsuitable for analysis (40). Although alterations in DNA damage and repair markers were observed in patients with early kidney disease, these findings were based on a single time-point assessment; longitudinal follow-up was not performed. It is worth conducting further studies that include patients with all 5 stages of CKD with follow-up every six months.

### 5.2 Strengths of the study

100 patients with early stages of CKD with T2D and 88 without T2D were included in the study. The marker 8-OHdG was determined to have increased levels in patients with T2D from stage 3 of CKD in T2D. The decrease in the DNA repair enzyme was observed in stages 2 and 3 in T2D. In the available scientific literature, we did not find any background on the expression of DNA damage and repair markers in the early stages of CKD in patients with and without T2D.

## Data Availability

The database that supports the conclusions of this research work will be made available by the authors, upon express request and with the authorization of the Ethics and Research Committee. Requests to access the datasets should be directed to the Research and Ethics Committee of the Civil Hospital of Guadalajara “Juan I. Manchaca”.
